# Structural insight into an evolutionarily ancient programmed cell death regulator – the crystal structure of marine sponge BHP2 bound to LB-Bak-2

**DOI:** 10.1038/cddis.2016.469

**Published:** 2017-01-12

**Authors:** Sofia Caria, Mark G Hinds, Marc Kvansakul

**Affiliations:** 1 Department of Biochemistry & Genetics, La Trobe Institute for Molecular Science, La Trobe University, Melbourne,Victoria 3086, Australia; 2 Department of Chemistry & Physics, La Trobe Institute for Molecular Science, La Trobe University, Melbourne, Victoria 3086, Australia

## Abstract

Sponges of the porifera family harbor some of the evolutionary most ancient orthologs of the B-cell lymphoma-2 (Bcl-2) family, a protein family critical to regulation of apoptosis. The genome of the sponge *Geodia cydonium* contains the putative pro-survival Bcl-2 homolog BHP2, which protects sponge tissue as well as mammalian Hek-293 and NIH-3T3 cells against diverse apoptotic stimuli. The Lake Baikal demosponge *Lubomirskia baicalensis* has been shown to encode both putative pro-survival Bcl-2 (LB-Bcl-2) and pro-apoptotic Bcl-2 members (LB-Bak-2), which have been implied in axis formation (branches) in *L. baicalensis.* However, the molecular mechanism of action of sponge-encoded orthologs of Bcl-2 remains to be clarified. Here, we report that the pro-survival Bcl-2 ortholog BHP2 from *G. cydonium* is able to bind the BH3 motif of a pro-apoptotic Bcl-2 protein, LB-Bak-2 of the sponge *L. baicalensis*. Furthermore, we determined the crystal structure of BHP2 bound to LB-Bak-2, which revealed that using a binding groove conserved across all pro-survival Bcl-2 proteins, BHP2 binds multi-motif Bax-like proteins through their BH3-binding regions. However, BHP2 discriminates against BH3-only bearing proteins by blocking access to a hydrophobic pocket that is critical for BH3 motif binding in pro-survival Bcl-2 proteins from higher organisms. This differential binding mode is reflected in a structure-based phylogenetic comparison of BHP2 with other Bcl-2 family members, which revealed that BHP2 does not cluster with either Bcl-2 members of higher organisms or pathogen-encoded homologs, and assumes a discrete position. Our findings suggest that the molecular machinery and mechanisms for executing Bcl-2-mediated apoptosis as observed in mammals are evolutionary ancient, with early regulation of apoptotic machineries closely resembling their modern counterparts in mammals rather than *Caenorhabditis elegans* or drosophila.

Sponges (*Porifera*) and Hydra (*Cnidaria*) are thought to be among the earliest metazoans, multicellular organisms that have differentiated tissues, yet they have evolved highly sophisticated mechanisms of homeostatic regulation.^1^ Sponge cells harbor a capacity for unlimited proliferation and differentiation potency,^2^ and as such a finely tuned control system necessary to maintain cellular homeostasis. Sequence analysis identified in the sponge orthologous genes to those in higher organisms that regulate apoptosis (programmed cell death).^3^

A critical group of proteins that regulate apoptosis are the B-cell lymphoma-2 (Bcl-2) family and these proteins^4^ regulate intrinsic (mitochondrial-mediated) apoptosis.^5^ The Bcl-2 family is divided into pro-survival and pro-apoptotic members, all of which share one or more of four conserved Bcl-2 homology (BH) motifs.^6^ Pro-survival Bcl-2 proteins including Bcl-2, Bcl-x_L_, Bcl-w, Mcl-1, A1 and Bcl-B block apoptosis, whereas the pro-apoptotic Bcl-2 members are necessary for initiation of cell death. The pro-apoptotic Bcl-2 proteins are further subdivided into two groups, those that only harbor a BH3 domain, which are termed the BH3-only proteins,^7^ and the multi-BH motif proteins Bax, Bak and Bok^8^ essential for cell death initiation. The BH3-only proteins including Bim, Puma, Bad and Noxa either directly activate pro-apoptotic Bax and Bak, or neutralize pro-survival Bcl-2 members by engaging their receptor binding groove using the *α*-helical BH3 domains.^9^ Upon activation, Bax and Bak oligomerize to cause organellar damage including permeabillization of the outer mitochondrial membrane,^10, 11^ thus releasing critical pro-apoptogenic factors such as cytochrome *c* and SMAC/Diablo from mitochondria activates caspases that destroy the cell.^12^ Detailed structural and binding studies have been performed on mammalian and worm apoptosis regulators showing many aspects of the mechanism of apoptosis are conserved across phyla.^13^

Evolutionary ancient ancestors of Bcl-2 proteins have been identified in sponges.^14^ The sponge *Geodia cydonium* expresses a putative pro-survival Bcl-2 homolog, BHP2, that protects sponge tissue against apoptosis induced by heat-shock or tributylin (an organic tin derivative) exposure, as well as mammalian Hek-293 and NIH-3T3 cells against serum withdrawal or tributylin.^15^ Similarly, the Lake Baikal demosponge *Lubomirskia baicalensis* harbors both putative pro-survival Bcl-2 (LB-Bcl-2) and pro-apoptotic Bcl-2 members (LB-Bak-2)^16^ that were postulated to be involved in axis formation (branches) in *L. baicalensis,* however their mechanism of action remains to be clarified. These findings indicate that the sponges maintain an apoptotic machinery to regulate homeostasis. To elucidate molecular mechanisms of apoptosis in these early lifeforms we have performed structural and binding studies on BHP2 and LB-Bak-2 from *G. cydonium* and *L. baicalensis*, respectively. We demonstrate that BHP2 shares the Bcl-2 fold with its higher organisms and, like them, bears a binding groove that binds the BH3-region of LB-Bak-2. Thus, mechanistically the sponge appears to replicate features of the mammalian rather than worm or fly apoptotic program and implies that the pro-apoptotic Bax-like proteins are regulated by interaction with the pro-survival Bcl-2 proteins in the sponge.

## Results

**Figure 1:**
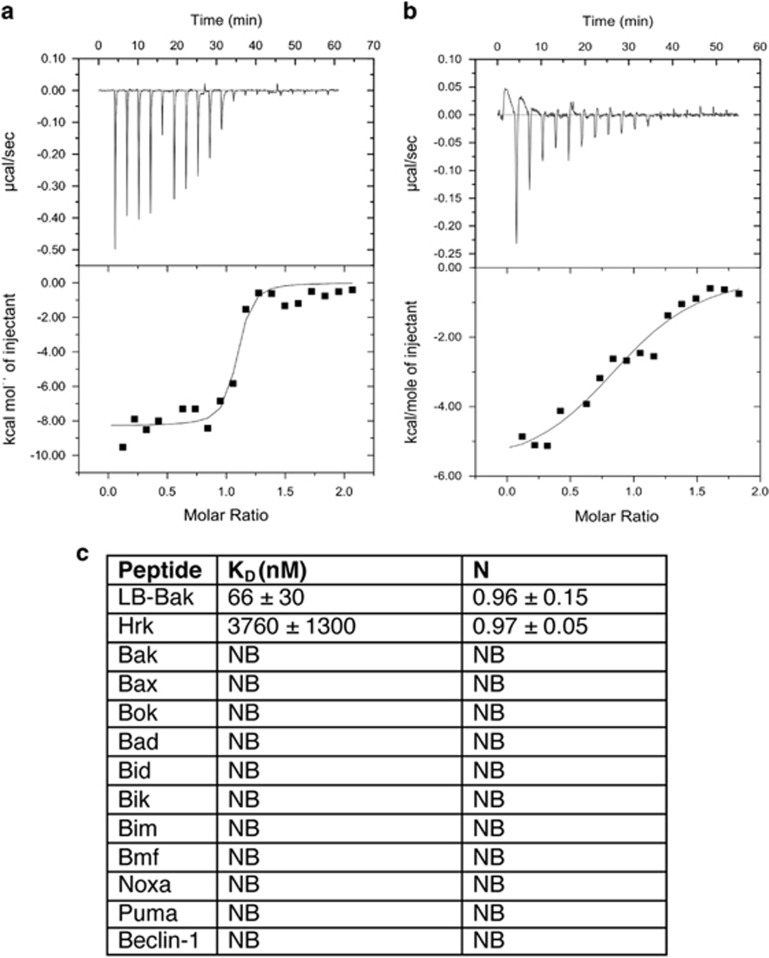
BHP2 interactions with BH3 domain peptides of pro-apoptotic Bcl-2 proteins. The affinity of recombinant BHP2 for BH3 domain peptides (26-mers, except for a Bax 28-mer and a Bid 34-mer) was assessed using isothermal titration calorimetry. *K*_D_ values (in nM) are the means of three experiments±S.D. NB, no binding detected, N denotes stoichiometry

To resolve the molecular mechanism underlying Bcl-2-mediated apoptosis in sponges, we examined the ability of the pro-survival Bcl-2 homolog BHP2 from *G. cydonium* to bind pro-apoptotic Bcl-2 proteins. Biochemical studies of a related pro-survival Bcl-2 protein from the demosponge *L. baicalensis* were also considered, however lack of expression ultimately precluded an analysis (data not shown). As pro-apoptotic Bcl-2 orthologs have not been identified in *G. cydonium,* we determined the capacity of BHP2 to bind a Bak-2 homolog from a related demosponge *L. baicalensis*, LB-Bak-2, or indeed any other human pro-apoptotic Bcl-2 family member. Using recombinant BHP2 we measured binding to peptides spanning the BH3 motif of LB-Bak-2, as well as all human BH3-only proteins and Bak, Bax and Bok. BHP2 bound LB-Bak-2 BH3 peptides with high affinity (*K*_D_=65 nM) (Figure 1a), whereas with the exception of Hrk (*K*_D_=3760 nM) (Figure 1b), no other BH3 motif of human pro-apoptotic Bcl-2 proteins displayed any significant binding (Figure 1c).

**Figure 2:**
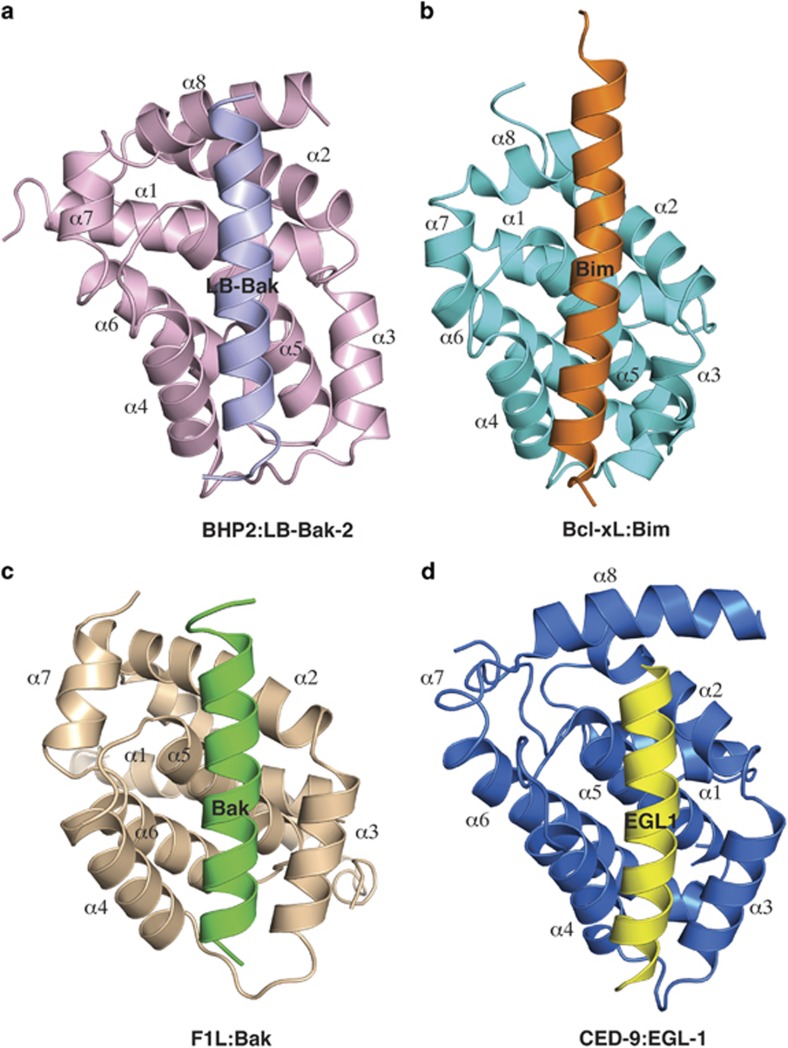
Crystal structure of BHP2 bound to LB-Bak-2 BH3 domain. (**a**) BHP2 (pink) in complex with the LB-Bak-2 BH3 domain (violet). BHP2 helices are labeled *α*1–8. The view in (**a**) is into the hydrophobic binding groove formed by helices *α*3–5. (**b**) Bcl-xL (cyan) in complex with the Bim BH3 domain (orange).^43^ The view is as in (**a**). (**c**) Vaccinia virus F1L (sand) in complex with the Bak BH3 domain (green).^45^ The view is as in (**a**). (**d**) CED9 (blue) in complex with the EGL1 BH3 domain (yellow).^44^ The view is as in (**a**).

**Figure 3:**
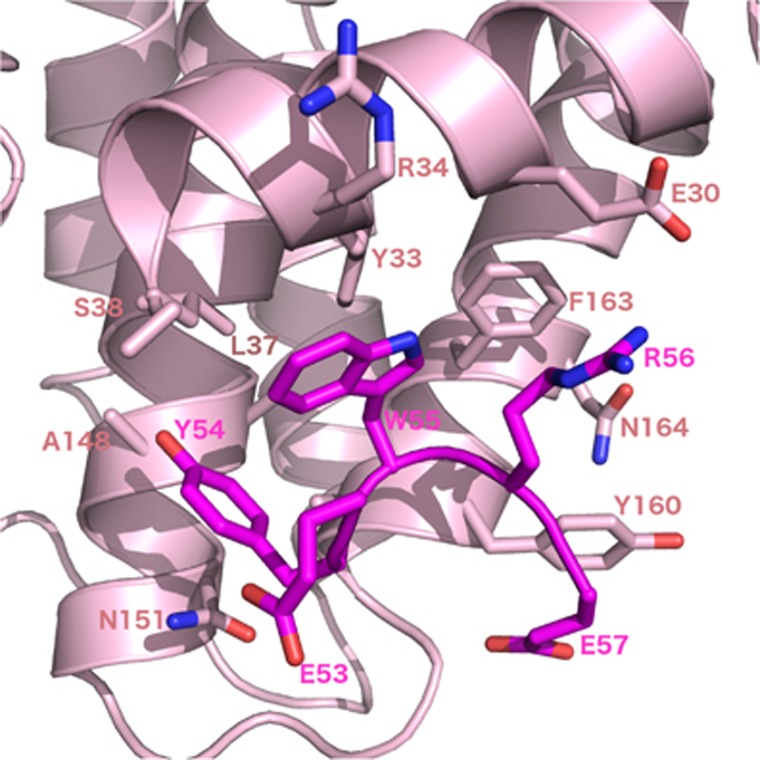
Detailed view of the helix turn in the BHP2 *α*1/*α*2 loop. BHP2 (pink) main chain is shown as cartoon. BHP2 residues comprising the ordered *α*1/*α*2 loop segment are shown as magenta

The structural basis for BH3 motif binding by BHP2, and its ability to discriminate against human pro-apoptotic Bcl-2 proteins over their sponge counterparts, was determined by solving the crystal structure of BHP2 bound to the LB-Bak-2 BH3 motif (Figure 2; Table 1). Like other Bcl-2 orthologs BHP2 adopts a globular helical bundle fold comprising 8 *α*-helices, with helices 2–5 forming the canonical binding groove observed in all other pro-survival Bcl-2 family members.^6^ Clear and continuous electron density is observed for residues 18–38 and 67–198, with the intervening linker region being largely disordered except for a short helical turn formed by residues 53–57 that anchor the loop to the solvent exposed side of helix 6 from the main globular motif (Figure 3). A DALI^17^ similarity analysis revealed that among all Bcl-2 proteins, complexes of Bcl-x_L_ (PDB ID 1PQ1) and Bcl-2 (PDB ID 5FCG) bound to BH3 domain peptides are most similar to BHP2, with r.m.s.d. values of 2.2 Å in both cases over 137 and 134 C*α* atoms, respectively.

**Figure 4:**
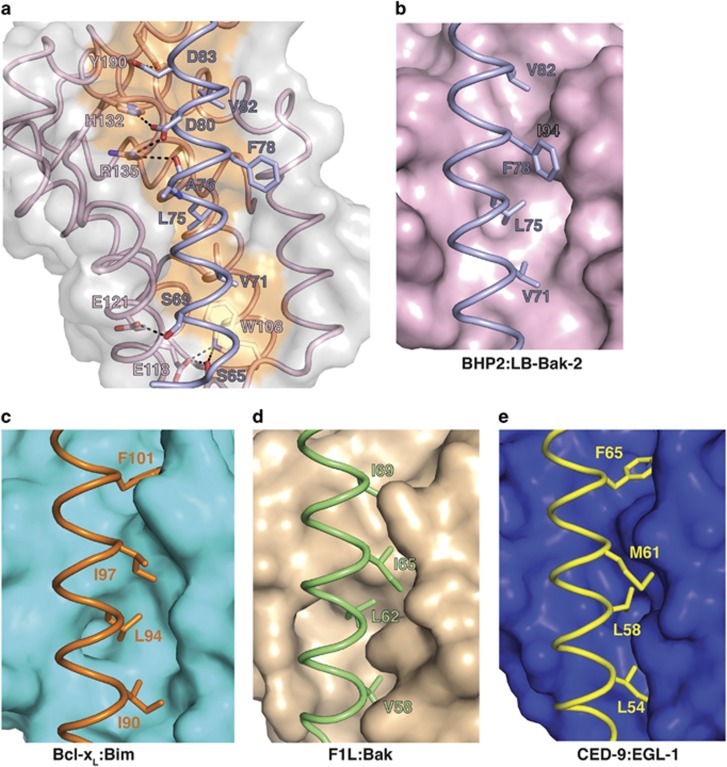
(**a**) Detailed view of the BHP2:LB-Bak-2 BH3 interface. The BHP2 surface, backbone and binding groove are shown in gray and pink respectively, whereas LB-Bak-2 BH3 is shown in violet. The four key hydrophobic residues of LB-Bak-2 (V71, L75, F78 and V82) on protruding into the binding groove, and the conserved salt bridge formed by LB-Bak D80 and BHP2 R135 are labeled, as well as residues involved in hydrogen bonds. Comparison of the P3 pocket among pro-survival Bcl-2: pro-apoptotic BH3 domain complexes. (**b**) BHP2:LB-Bak-2 complex. (**c**) Bcl-x_L_:Bim complex. (**d**) CED9:EGL1 complex. (**e**) F1L:Bak complex

Similar to pro-survival Bcl-2 proteins from higher organisms, BHP2 engages the LB-Bak-2 BH3 motif via the canonical Bcl-2 binding groove formed by *α*-helices 2–5. A total of 913 Å^2^ of solvent accessible surface is buried in the process. As expected, BHP2 Arg135^BHP2^ from the conserved BH1 motif forms an ionic interaction with the LB-Bak-2 Asp80^Bak^, an interaction conserved in almost all Bcl-2-BH3 motif interactions. Interestingly, Asp80^Bak^ is also engaged in a second ionic interaction with His132^BHP2^, which in turn also contacts Asp83^Bak^, leading to the formation of a network of ionic interactions centering on LB-Bak-2 Asp80. In addition, the BHP2:LB-Bak-2 interface features several hydrogen bonds between Glu118^BHP2^ and Ser69^Bak^, Tyr190^BHP2^ and Asp83^Bak^, Arg135^BHP2^ and Ala76^Bak^, and Glu118^BHP2^ and Trp108^BHP2^ with Ser65^Bak^. A hallmark of pro-survival Bcl-2:pro-apoptotic BH3 motif interactions is the engagement of four highly conserved hydrophobic residues from the BH3 motif by the ligand binding groove of pro-survival Bcl-2.^7^ While BHP2 accommodates V71, L75 and V82, unexpectedly F78 is not captured by a hydrophobic pocket at the floor of the binding grove. Instead, the helical turn in LB-Bak-2 BH3 that features F78 is bulged out, allowing F78 to sit on top of BHP2 I94 (Figure 4a). This shift is enforced by the positioning of I94 in BHP2, where the protruding Ile side chain prevents access of LB-Bak-2 F78 into the typical position adopted in other pro-survival Bcl-2:BH3 motif complexes (Supplementary Figure S2).

**Figure 5:**
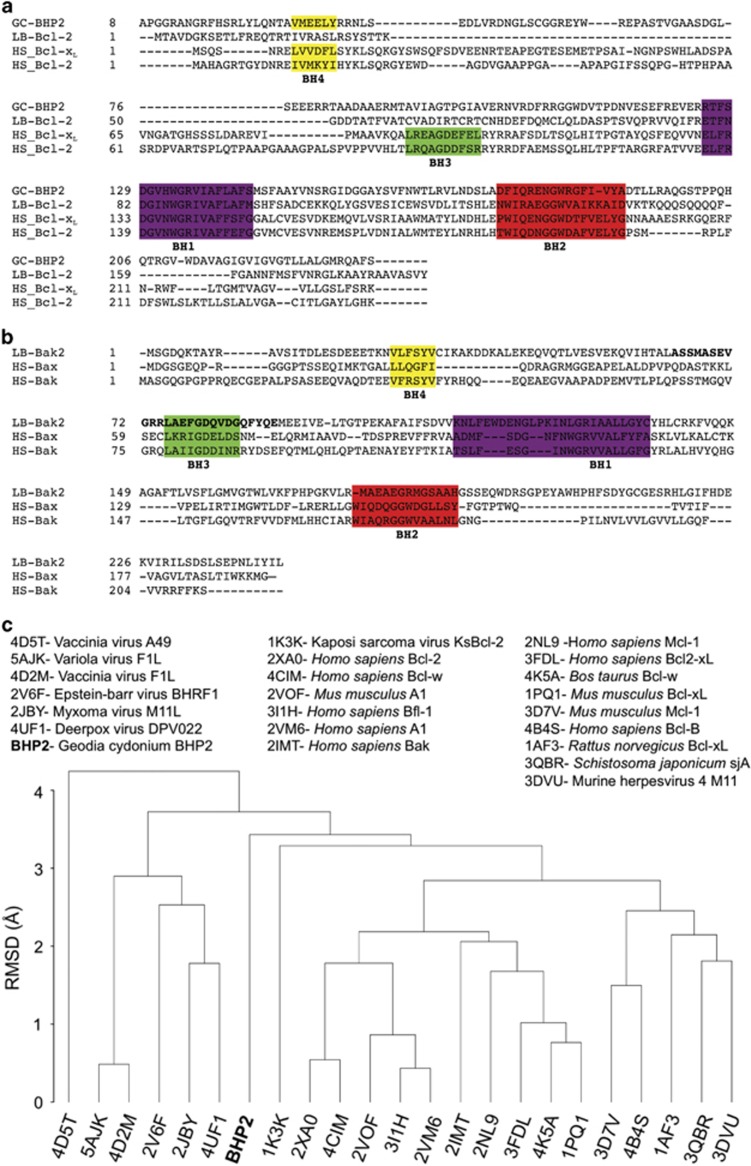
(**a**) Sequence alignment of BHP2 with pro-survival Bcl-2 family members. (**b**) Sequence alignment of BH3 domains of LB-Bak-2 and human pro-apoptotic Bcl-2 proteins. (**c**) Structure-based phylogenetic analysis of pro-survival Bcl-2 proteins of known structure

To further understand the evolutionary relationship between BHP2 and Bcl-2 family proteins from higher organisms, we performed a structure-guided evolutionary analysis using pro-survival Bcl-2 family proteins of known structure by performing a pairwise comparison of experimentally determined structures of pro-survival Bcl-2 proteins using r.m.s.d. values for equivalent C*α* atoms. We found that BHP2 did not cluster with other Bcl-2 proteins, with family members originating from higher organisms forming one cluster and pathogen-encoded family members forming a second cluster (Figure 5c).

## Discussion

Bcl-2 proteins are the major arbiter of intrinsic apoptosis in higher organisms regulating the release of apoptogenic factors through the mitochondrial outer membrane.^6^ Essentially, there are two subgroups of the Bcl-2 family, the folded Bcl-2-like proteins which are either pro-survival (Bcl-2, Bcl-x_L_, Mcl-1, A1 and Bcl-B or pro-apoptotic (Bak, Bax, Bok, the latter two appear later in evolution)), and a phylogenetically distinct pro-apoptotic BH3-only family (Bim, Bad, Bmf, tBid, Bik, Noxa, Puma, Hrk, in mammals) that are unstructured.^7^ However, there are species-specific differences in their mode of action and it is not clear if the BH3-only proteins exist in all phyla.^18^ Despite the identification of ancient sequence orthologs of Bcl-2 family members in demosponges (Figures 5a and b)^15, 16^ and evidence that sponge Bcl-2 orthologs are able to inhibit apoptosis,^15^ it remained unclear if such ancient Bcl-2 proteins function mechanistically in a manner analogous to their modern counterparts.

BHP2 is able to protect HEK-293 cells against apoptosis under cell culture conditions,^15^ however, the molecular basis for this apoptosis inhibition in a human cell line remains to be understood. We find that BHP2 is able to bind the BH3 motif of the *L. baicalensis* pro-apoptotic Bcl-2 homolog LB-Bak-2 with high affinity. However, only low affinity to Hrk BH3 and no binding to other BH3 peptides from human Bcl-2 proteins including those from human Bak, Bax or BH3-only proteins was measured. Consequently, there is no obvious mechanism for BHP2 to inhibit intrinsic apoptosis in human cell lines. However, a similar situation was observed when human Bcl-2 was overexpressed in *C. elegans.*^19, 20^ Bcl-2 potently protected against apoptosis in the worm, despite its low affinity for pro-apoptotic Egl1 (*K*_D_=7.1 *μ*M).^21^

The crystal structure of the BHP2:LB-Bak-2 BH3 complex demonstrates BHP2 utilizes the conserved canonical binding groove found in all other pro-survival Bcl-2 family members to date. Despite the overt mechanistic similarity with mammalian Bcl-2 proteins there are key differences in how BHP2 engages BH3 motifs. BHP2 engagement of the LB-Bak-2 BH3 motif relies only on the use of three conserved hydrophobic residues in the BH3 domain, with the third hydrophobic residue F78^Bak^ being pushed out of the BHP2 ligand binding groove and accommodated via a pronounced bulge in the otherwise helical LB-Bak-2 BH3 motif. The unexpected absence of a pocket suitable to accommodate the LB-Bak-2 F78 residue is likely to be a major structural determinant for the selectivity of BHP2 for BH3 motif ligands. Indeed, of all human pro-apoptotic BH3 motifs tested, only Hrk revealed a modest affinity of 3.7 *μ*M for BHP2, with the BH3 motif of human Bak displaying no detectable binding.

Interestingly, a major difference between LB-Bak-2 and human Bak BH3 motifs centers on LB-Bak-2 F78, the equivalent residue in human Bak is an isoleucine (I81) that is unable to rotate in the manner that allows the corresponding phenylalanine in LB-Bak-2 to swing out of the way to minimize steric hindrance. The observed differences in the mode of engagement of a BH3 motif with BHP2 compared with other pro-survival Bcl-2 proteins that utilize four pockets to bind BH3 motifs is also reflected in the structure-based phylogenetic analysis of BHP2 (Figure 5c), which suggests that BHP2 is unique when compared with mammalian- and pathogen- encoded pro-survival Bcl-2 proteins.

Our findings provide a mechanistic platform for understanding apoptosis regulation by early Bcl-2 orthologs. Apoptotic features in sponges were first observed in hibernating sponges and like higher metazoans, they have also been observed during developmental processes.^2, 22^ Furthermore, dysregulated apoptosis in these simple organisms is a potential mechanism for disease. Surprisingly, it has been observed that like humans, Hydra are susceptible to tumors where deregulated apoptosis appears to be the underlying cause.^23^ However, the molecular underpinnings of these observations have not yet been elucidated.

The presence of functional pro-survival Bcl-2 and pro-apoptotic Bak suggests that sponges may also harbor one or more proteins bearing pro-apoptotic BH3 motif sequences to act in a manner analogous to BH3-only proteins in higher organisms, as indeed has been identified in hydra.^24^ However, to date no such sponge BH3-only protein has been identified, it may simply be that the BH3 sequence in these proteins lacks sufficient similarity to be recognized, as the BH3 motif is only a short motif with little absolute conservation.^7^ Alternatively, the BH3-only proteins may have evolved later in evolutionary time. The presence of a Bak-like molecule that can be engaged by a pro-survival BHP2 suggests that apoptosis regulation in sponges is more similar to what is found in mammals compared with, for example, the worm *Caenorhabditis elegans*, where Bax/Bak orthologs are absent and CED-4 fulfils the role assumed by Apaf-1 in mammals to control caspase activation.^25^ However, it is noted that *C. elegans* may be unusual among the worms, with Platyhelminthes^26^ harboring intrinsic apoptotic machineries that resemble their more modern mammalian relatives.

In summary, our findings suggest that the molecular machinery and mechanisms for executing Bcl-2-mediated apoptosis as observed in mammals are evolutionary ancient, with early regulation of apoptotic machineries closely resembling their modern counterparts in mammals rather than *C. elegans* or drosophila.

## Materials and methods

### Cloning, expression and purification

The codon-optimized cDNA of BHP2 (Uniprot accession number Q967D2) encoding for residues 18–200 was synthesized (GenScript, Piscataway, NJ, USA) and cloned into the pGEX-6P1 vector and expressed in *Escherichia* *coli* BL21 (DE3) star cells. Cells were grown in 2YT auto induction medium^27^ containing 1 mg/ml ampicillin at 25 °C in a shaker incubator operating at 160 rev/min for ~24 h. The cells were harvested by centrifugation at 6000 rev/min (JLA 9.1000 rotor, Beckman Coulter Avanti J-E, Beckman Coulter, Mount Waverley, VIC) for 20 min and resuspended in 50 ml lysis buffer A (50 mM Tris pH 8.5, 150 mM NaCl and 1 mM ethylene diamine tetraacetic acid (EDTA)) supplemented with lysozyme. The cells were lysed using one cycle at 35 kPi's in the benchtop TS cell disrupter (Constant Systems Ltd, Daventry, UK). The lysate was transferred to SS34 tubes for further centrifugation at 16 000 rev/min (JA-25.50 rotor, Beckman Coulter Avanti J-E) for 20 min. The supernatant loaded onto a 2 ml Glutathione Sepharose 4B resin column (GE Healthcare, Aurora, OH, USA) equilibrated with buffer A. After sample application, the column was washed with five column volumes of buffer A followed by HRV 3C protease cleavage on column overnight at 4 °C.

The column was washed with five column volumes of buffer A to remove the liberated target protein and concentrated to a volume of 1 ml. Concentrated BHP2 was subjected to size-exclusion chromatography using a Superdex S200 10/300 column mounted on an ÄKTApure system (GE Healthcare) equilibrated in 25 mM HEPES pH 7.5, 150 mM NaCl, where it eluted as a single peak. The final sample purity was estimated to be higher than 95% based on SDS-PAGE analysis.

### Measurement of dissociation constants

Binding affinities were measured using a MicroCal iTC200 system (GE Healthcare) at 25 °C using BHP2 in 25 mM HEPES pH 7.5, 150 mM NaCl at a final concentration of 20 *μ*M. BH3 domain peptide ligands were used at a concentration of 200 *μ*M and titrated using 19 injections of 2.0 *μ*l of ligand. All affinity measurements were performed in triplicate. Protein concentrations were measured using a Nanodrop UV spectrophotometer (Thermo Fisher Scientific, Scoresby, Victoria, Australia) at a wavelength of 280 nm. Peptide concentrations were calculated based on the dry peptide weight after synthesis. The BH3 domain peptides used were commercially synthesized using liquid-phase peptide synthesis (GenScript) and were purified to a final purity of 95%. With the exception of LB-Bak-2 BH3 all peptide sequences were previous described.^28^ LB-Bak-2 BH3 peptides span residues 62–89 (Uniprot accession code Q1RPT5).

### Crystallization and data collection

The complex of BHP2 with LB-Bak-2 BH3 was reconstituted as previously described^29^ by adding LB-Bak-2 BH3 domain peptide at a 1:1.25 M ratio to BHP2. The reconstituted complex was concentrated to 5 mg/ ml using a 3 kDa molecular weight cut-off centrifugal concentrator (Millipore, Bayswater, Victoria, Australia), flash-cooled and stored under liquid nitrogen. High-throughput sparse matrix screening was carried out using 96-well sitting-drop trays (Swissci, Neuheim, Switzerland) and the vapor diffusion method at 20 °C at the CSIRO Collaborative Crystallization Centre, Melbourne, Australia. The initial crystallization conditions used were from commercially available screening kits (Shotgun Screen, PACT Com7 C3 and JCSG-C3 from C3 CSIRO).

Crystals of BHP2:LB-Bak-2 BH3 were obtained at a protein concentration of 5 mg/ml using the sitting-drop method at 20 °C in 28.7% (w/v) PEG 1500, 0.1 M bis-Tris chloride (Supplementary Figure S1b). The crystals were flash-cooled at −173.15 °C in mother liquor supplemented with 20% (v/v) ethylene glycol. The BHP2:LB-Bak-2 BH3 complex forms clusters of plate-shaped crystals belonging to space group I2 in the monoclinic crystal system.

Diffraction data were collected on the MX2 beamline at the Australian Synchrotron using a ADSC Quantum 315r CCD detector (Area Detector Systems Corporation, Poway, CA, USA) with an oscillation range of 1.0° per frame and a wavelength of 0.9537 Å. Diffraction data were integrated using XDS^30^ and scaled using AIMLESS.^31^ Crystals of BHP2:LB-Bak-2 BH3 contained two chains of BHP2 and two chains of LB-Bak-2 BH3 in the asymmetry unit, with a calculated solvent content of 45.73%. The structure was phased by molecular replacement using MrBUMP^32^ using Bcl-w as a search model (PDB accession code 4k5b^33^) and an initial model was built using PHENIX Autobuild^34^ with Resolve and Buccaneer.^35^ The final BHP2:LB-Bak-2 complex was manually built using Coot^36^ and refined using PHENIX.^37^ Details of the data collection and refinement statistics are summarized in Table 1. All images were generated using PyMOL (New York, USA). All software was accessed via SBGrid.^38^

### R.m.s.d. cluster analysis

The structures of Bcl-2 protein family members were selected from the Protein Data Bank for structural comparison, with one representative structure selected for proteins with multiple experimentally determined structures. Structure-based sequence alignment was performed in STRAP.^39^ The sequence alignment and superimposed coordinates of the different models were loaded into to R program^40^ using the bio3d package^41^ for an r.m.s.d. cluster analysis.

### Availability of data and material

Coordinate files have been deposited in the Protein Data Bank under the accession codes 5TWA. Raw diffraction images were deposited in the SBGrid Data Bank^42^ using the accession code doi:10.15785/SBGRID/388.

## Figures and Tables

**Table 1:** X-ray data collection and refinement statistics

Space group	I2
	
*Unit-cell parameters*	
a (Å)	68.31
b (Å)	51.59
c (Å)	107.80
*α*=*γ* (°)	90.00
*β* (°)	96.34
Wavelength (Å)	0.95370
Resolution (Å)	41.08–1.85 (1.89–1.85)
*R*_merge_	0.13 (1.08)
*R*_pim_	0.10 (0.79)
<I/σ(I)>	8.10 (1.60)
CC1/2	1.00 (0.56)
Completeness (%)	99.70 (99.90)
Multiplicity	5.00 (5.00)
No. of unique reflections	31 937 (1987)
No. of observed reflections	158 961 (9860)
	
*Refinement*	
Resolution range (Å)	33.95–1.85 (1.92–1.85)
Reflections (working set/test set)	31 911/1587
Protein atoms	2969
Solvent molecules	74
*R*_work_/*R*_free_	0.1962/0.2377
r.m.s.d., angles (°)	1.31
r.m.s.d., bonds (Å)	0.02
	
*Ramachandran plot (%)*	
Favored	99.0
Allowed	1.1
Disallowed	0.0

Values in parentheses are the values for the highest resolution shell
