# Supplementary figures and images for: Structural insight into an evolutionarily ancient programmed cell death regulator – the crystal structure of marine sponge BHP2 bound to LB-Bak-2
Source: Cell Death Dis. 2017 Jan 12;8(1):e2543–. doi: 10.1038/cddis.2016.469 (PMC5386376; doi:10.1038/cddis.2016.469)

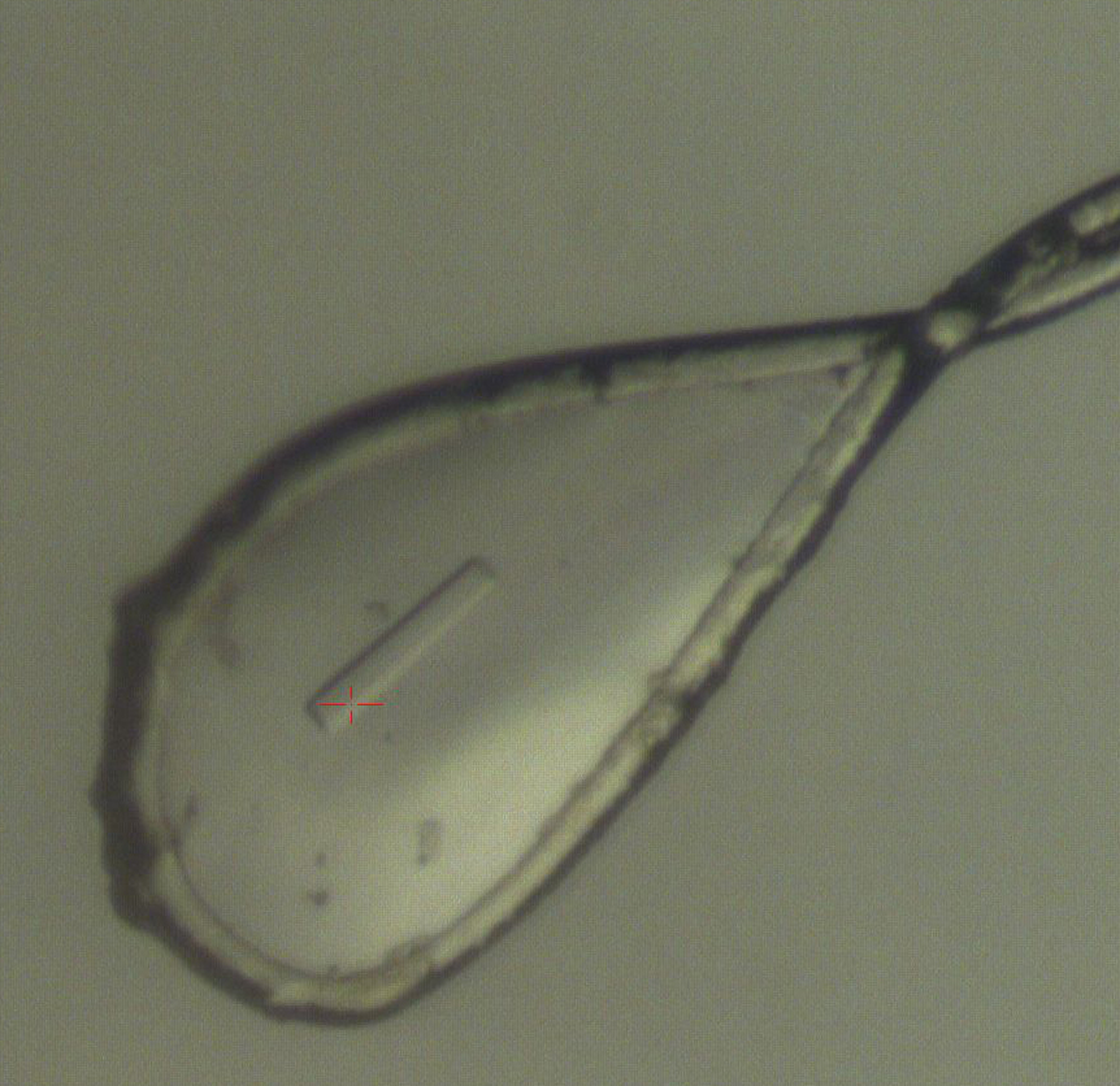

Supplement: Supplementary Figure S1 [file cddis2016469x1.tif]

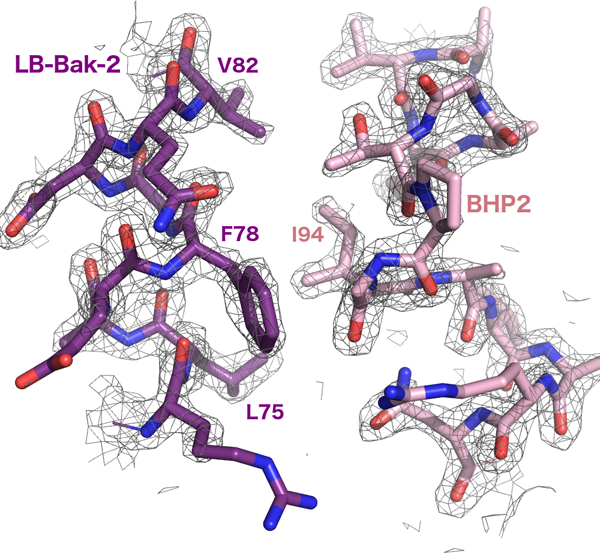

Supplement: Supplementary Figure S2 [file cddis2016469x2.tif]
